# Quality, reliability, and user engagement of hepatitis B–related short videos on TikTok, Bilibili, and Xiaohongshu: a cross-platform cross-sectional study

**DOI:** 10.3389/fdgth.2026.1880030

**Published:** 2026-10-09

**Authors:** Lili Yu, Hongyu Wei, Yuanyuan Zhang

**Affiliations:** 1 School of Nursing, Guangxi University of Traditional Chinese Medicine, Nanning, China; 2 The Second Affiliated Hospital of Guangxi University of Chinese Medicine, Nanning, China

**Keywords:** health information quality, hepatitis B, patient education, short video platforms, social media

## Abstract

**Background:**

CHB remains a major public health burden in China. TikTok, Bilibili, and Xiaohongshu have become key channels for health information, yet the quality, reliability, and transparency of such content remain uncertain.

**Methods:**

On November 12, 2025, the top 100 eligible HBV-related videos from each platform (*N* = 300) were assessed using the Global Quality Scale (GQS), modified DISCERN instrument (mDISCERN), and Journal of the American Medical Association benchmark criteria (JAMA). Two raters independently scored each video; discrepant ratings were resolved by consensus or senior adjudication, and final consensus scores were used for the main analyses. Inter-rater agreement was assessed using linearly weighted Cohen's *κ*. Group differences were evaluated using the Kruskal–Wallis H test followed, when significant, by Dunn's *post hoc* test with Holm adjustment. Spearman correlations and multivariable ordinal regression models were used to examine unadjusted and adjusted associations.

**Results:**

A total of 300 videos were included, with 100 from each platform. Inter-rater agreement was substantial for GQS (*κ* = 0.774, 95% CI 0.705–0.839), almost perfect for mDISCERN (*κ* = 0.928, 95% CI 0.888-0.962), and moderate for JAMA (*κ* = 0.503, 95% CI 0.410-0.590). mDISCERN and JAMA scores differed significantly across platforms, whereas GQS scores did not. Medical personnel had higher rank distributions than normal users for GQS, mDISCERN, and JAMA after Holm adjustment. In adjusted models, Xiaohongshu was associated with higher GQS scores than Bilibili, normal-user uploaders were associated with lower GQS scores than medical personnel, and longer video duration was associated with higher GQS scores. JAMA showed threshold-specific associations with platform, uploader type, and video duration. The adjusted cumulative mDISCERN model was not formally interpreted because of sparse-data instability; a bias-reduced adjacent-category model was reported as an exploratory sensitivity analysis. Engagement metrics showed weak and inconsistent correlations with quality scores.

**Conclusions:**

Among highly visible hepatitis B-related short videos retrieved under prespecified search conditions, video quality varied by assessment instrument, platform, and uploader type. Adjusted associations were instrument- or threshold-specific, and engagement metrics should not be treated as proxies for reliability or transparency.

## 1 Introduction

Chronic hepatitis B virus (HBV) infection remains a major global and Chinese public health burden. HBV can cause chronic liver disease, cirrhosis, and hepatocellular carcinoma, and many infected people remain undiagnosed or are not linked to appropriate care (1, 2). Recent epidemiological evidence indicates that China still has a substantial burden of chronic HBV infection, although prevalence has changed over time with vaccination and prevention efforts (3, 4).

Digital media and short-video platforms have become important channels for health information seeking and public health communication. Social media can expand the reach of health messages (5), and TikTok has been identified as a source from which users obtain health information and misinformation (6). However, objective assessments and reviews of health-related short videos have shown that accessibility and popularity do not necessarily ensure scientific reliability or transparency (7–9). These concerns are particularly relevant for HBV because prevention, testing, treatment eligibility, and long-term monitoring require medically accurate explanations.

Previous research has documented broader risks related to health misinformation and infodemics online (10, 11). At the same time, social media-based interventions have been used to promote HBV screening and liver cancer prevention in specific communities (12). Studies evaluating short videos on other health topics have reported variable quality and reliability across platforms and diseases (13–15). However, evidence remains limited on HBV-related short videos across multiple Chinese-language platforms, and other-disease findings cannot be assumed to apply directly to HBV.

Bilibili's 2024 annual report describes the platform as a video community with a strong content ecosystem and knowledge-sharing functions (16). This platform background supports the rationale for cross-platform comparison, but it should not be interpreted as evidence that any platform produces higher-quality medical information.

Therefore, this study quantitatively evaluated the content characteristics, scientific quality, and reliability of HBV-related short videos on TikTok, Bilibili, and Xiaohongshu. We also examined the relationship between video quality and user engagement. These findings may provide evidence for improving HBV-related health communication on social media and strengthening public awareness of HBV prevention and control.

## 2 Methods

### 2.1 Ethical considerations

This study analyzed publicly available short-video content and publicly accessible account-level information from Xiaohongshu, Bilibili, and TikTok. The researchers did not interact with users or access private, sensitive, or non-public data. Identifiable account names and video URLs are not reported in this manuscript. Because the study was limited to publicly available online content and did not involve interaction with users or access to private data, no additional informed consent was required.

### 2.2 Search strategy and data collection

To reduce but not eliminate the influence of personalized recommendation algorithms, new accounts with no prior search or viewing history were created for each platform before data collection. Searches were conducted on Xiaohongshu, TikTok, and Bilibili on November 12, 2025, and all searches were completed within 4 h. Two Chinese search terms were used separately on each platform: “乙型肝炎” (“hepatitis B”) and “乙型病毒性肝炎” (“viral hepatitis B”). Search results were reviewed according to the default ranking provided by each platform because highly ranked videos are more likely to represent the information encountered by normal users.

For Xiaohongshu and TikTok, the first 200 ranked search results were reviewed sequentially, and the first 100 videos meeting the prespecified eligibility criteria were retained. Screening stopped once the target sample of 100 eligible videos per platform had been reached. At the time of data collection, 100 Bilibili videos were retrieved and retained. These videos were subsequently verified against the same eligibility criteria, and all were confirmed to be eligible. The final sample comprised 300 videos, with 100 videos from each platform.

Videos were excluded if they were unrelated to hepatitis B, duplicated within or between the two search results, primarily commercial advertisements, or uploaded within 7 days before the search date. When the same video appeared in the results for both search terms, it was retained only once. A total of 300 videos were included, comprising 100 videos from each platform (Figure 1).

**Figure 1:**
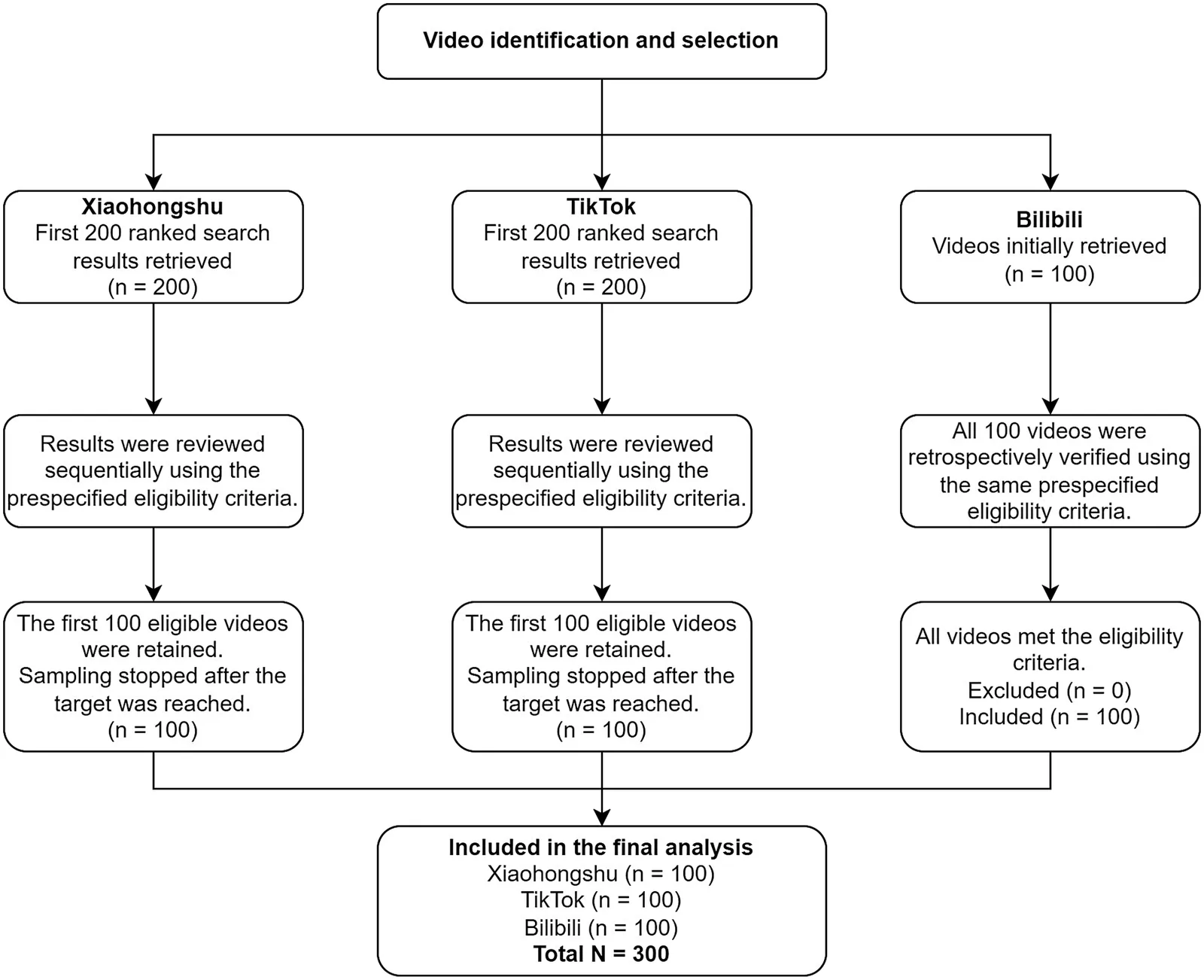
Platform-specific video identification and selection process.

Search results were ordered using each platform's default ranking system. The exact weighting of textual relevance, recency, engagement, uploader characteristics, geographic location, device or network information, and other ranking signals was not publicly available and could not be standardized across platforms. Creating new accounts reduced personalization based on prior viewing and search histories, but it could not eliminate cold-start recommendations, platform-specific ranking rules, temporal variation, geographic effects, or possible nondeterministic variation in search results. The retrieved videos should therefore be interpreted as a cross-sectional sample of highly visible results returned under the prespecified search conditions, rather than as a probability sample or exhaustive representation of all hepatitis B-related videos available on each platform.

### 2.3 Data extraction and classification

The following information was extracted for each video on the search date: platform, video URL, uploader identity, number of likes, collections, comments, and shares, uploader follower count, video duration, upload date, and days since upload. Likes, collections, comments, and shares were defined as user engagement indicators. Follower count was treated as an uploader-level characteristic, whereas video duration and days since upload were treated as structural video characteristics. The platform-specific save or favorite function was harmonized as “collections” for cross-platform analysis. All engagement data were recorded on the same date to reduce variation caused by the continuous accumulation of user interactions.

Uploader identity was classified into four mutually exclusive categories according to the account profile, platform verification information, and stated institutional affiliation:

Medical personnel: individual accounts identified as physicians, nurses, pharmacists, or other licensed or verifiable healthcare professionals;

Normal users: individual accounts without a verifiable healthcare professional identity or official medical institutional affiliation, including patients, caregivers, and members of the general public;

Medical institutions: official accounts operated by hospitals, clinics, public health institutions, or other healthcare facilities; and

Other organizations: accounts operated by nonmedical organizations, media organizations, nonprofit organizations, social organizations, or commercial enterprises.

When uploader identity was unclear, the account profile and platform verification information were jointly reviewed by the two evaluators, and disagreements were resolved through discussion with the senior evaluator.

Video content was coded using five non-mutually exclusive topic categories:

Symptoms: clinical manifestations, signs, or symptoms associated with HBV infection;

Causes: HBV etiology, transmission routes, risk factors, and mechanisms of infection;

Prevention: vaccination, screening, prevention of mother-to-child transmission, infection-control measures, and other preventive behaviors;

Treatment: antiviral treatment, treatment indications, medication use, clinical monitoring, and disease management; and

Prognosis: disease progression, long-term outcomes, complications, cirrhosis, and hepatocellular carcinoma.

Each topic was coded independently as present or absent. A single video could be assigned to more than one topic; therefore, the percentages across the five topic categories did not necessarily sum to 100%.

### 2.4 Assessment of video content and quality

Each video was independently assessed by two clinical experts using the GQS, mDISCERN, and JAMA benchmark criteria. Identical ratings were directly retained as the final score. Discrepant ratings were reviewed by the two raters to reach consensus; unresolved discrepancies were adjudicated by a senior reviewer. All three evaluators were frontline clinicians from tertiary hospitals, each with more than 13 years of clinical experience. The final consensus scores were used in the main analyses, whereas the original independent ratings were retained for assessment of inter-rater agreement.

The GQS is a 5-point scale used to evaluate the overall educational quality, flow, usefulness, and comprehensiveness of health information, with scores ranging from 1 to 5 (17). The mDISCERN was based on the original DISCERN framework and on the five-item modified DISCERN approach used for evaluating online medical videos, which assesses clarity of aims, reliability of sources, balance and bias, provision of additional sources, and acknowledgement of uncertainty (18, 19). The JAMA benchmark criteria include four items: authorship, attribution, disclosure, and currency; each item is scored as 1 for yes and 0 for no, giving a total score ranging from 0 to 4 (20). Higher scores indicate better quality, reliability, or transparency according to the corresponding instrument. Detailed items and scoring criteria are provided in Supplementary Table S1.

### 2.5 Statistical analysis

Continuous variables were summarized as medians and interquartile ranges because of their non-normal distributions. Inter-rater agreement for the ordinal total scores was assessed using linearly weighted Cohen's *κ*, calculated for the overall sample and separately for each platform. Exact agreement was also reported. The 95% confidence intervals for *κ* were estimated using 5,000 nonparametric bootstrap resamples; overall bootstrap resampling was stratified by platform. Continuous video characteristics and engagement indicators were compared across the three platforms using the Kruskal–Wallis H test because of their non-normal distributions. Binary video-topic indicators were compared across platforms using Pearson's chi-square test. All tests were two-sided. Differences in the final consensus GQS, mDISCERN, and JAMA scores across uploader types, platforms, and presentation formats were assessed using the Kruskal–Wallis H test. When the overall Kruskal–Wallis test was statistically significant, Dunn's *post hoc* pairwise comparisons were performed, with *P* values adjusted using the Holm method within each grouping-variable and assessment-instrument combination. Epsilon-squared values were calculated as effect-size estimates for the Kruskal–Wallis tests. Spearman rank correlation analyses were used to examine unadjusted associations between final consensus quality scores and likes, collections, comments, shares, video duration, days since upload, and follower count. Correlations were calculated using pairwise complete observations and were not interpreted as evidence of independent prediction or causality. Separate multivariable ordinal regression models were fitted for the final consensus GQS, mDISCERN, and JAMA scores. Core models included platform, uploader type, follower count, video duration, and days since upload. Follower count, video duration, and days since upload were transformed using log2(x + 1), so the corresponding odds ratios represented the approximate change associated with a doubling of each variable. Bilibili and medical personnel served as reference categories. For GQS and JAMA, predictors satisfying the proportional-odds assumption were modeled using common effects, whereas predictors with non-proportional effects were allowed to vary across cumulative score thresholds in partial proportional-odds cumulative logit models. For mDISCERN, uploader type violated the proportional-odds assumption, and a stable cumulative partial proportional-odds model could not be obtained because of sparse observations in some uploader and score-category combinations. A mean bias-reduced adjacent-category ordinal logistic regression was therefore conducted as an exploratory sensitivity analysis. Extended models additionally adjusted for five video-topic indicators: symptoms, cause, prevention, treatment, and prognosis. Models were evaluated for convergence, coefficient finiteness, covariance or Hessian stability, multicollinearity, and sparse-data problems. All tests were two-sided, and P < 0.05 was considered statistically significant.

## 3 Results

### 3.1 Video characteristics

A total of 300 hepatitis B-related short videos were included, with 100 videos from each platform: Bilibili, TikTok, and Xiaohongshu. Basic video characteristics and engagement indicators differed significantly across platforms (all *P* < 0.05). TikTok showed the highest user engagement. The median numbers of likes, collections, comments, and shares on TikTok were 1,685, 555, 291, and 292, respectively, which were higher than those on Bilibili (81, 72, 10, and 38) and Xiaohongshu (33, 23, 4, and 12) (Table 1 and Figure 2).

**Table 1:** Comparison of video characteristics and engagement indicators across platforms.

Variables	Total (*n* = 300)	Bilibili (*n* = 100)	TikTok (*n* = 100)	Xiaohongshu (*n* = 100)	P
Likes, M (Q1, Q3)	151 (29, 1,236)	81 (23,588)	1,685 (181,3,786)	33 (9,165)	<0.001
Collections, M (Q1, Q3)	79 (15, 476)	72 (16,305)	555 (64,1,792)	23 (6,128)	<0.001
Comments, M (Q1, Q3)	21 (2, 225)	10 (2.00,78.50)	291 (26,829)	4 (0.00,30.50)	<0.001
Shares, M (Q1, Q3)	43 (10, 283)	38 (12,144)	292 (27,830)	12 (3,64)	<0.001
Video duration, s, M (Q1, Q3)	99 (63, 190)	203 (100,297)	86 (53,134)	74 (47,122)	<0.001
Days since upload, M (Q1, Q3)	253 (132, 833)	1,204 (511,1,688)	182 (127,224)	211 (55,441)	<0.001
Followers, M (Q1, Q3)	10,370 (2130, 63,498)	6,266 (530,76,750)	39,894 (12199,109,115)	3,684 (1324,12,701)	<0.001
Video topic, *n* (%)					
Symptoms	78 (26.00)	24 (24.00)	24 (24.00)	30 (30.00)	0.536
Causes	78 (26.00)	48 (48.00)	27 (27.00)	3 (3.00)	<0.001
Prevention	169 (56.33)	66 (66.00)	56 (56.00)	47 (47.00)	**0**.**025**
Treatment	192 (64.00)	58 (58.00)	60 (60.00)	74 (74.00)	**0**.**037**
Prognosis	60 (20.00)	25 (25.00)	16 (16.00)	19 (19.00)	0.269

Videos could address more than one topic; therefore, the percentages of topic categories do not necessarily sum to 100%.

**Figure 2:**
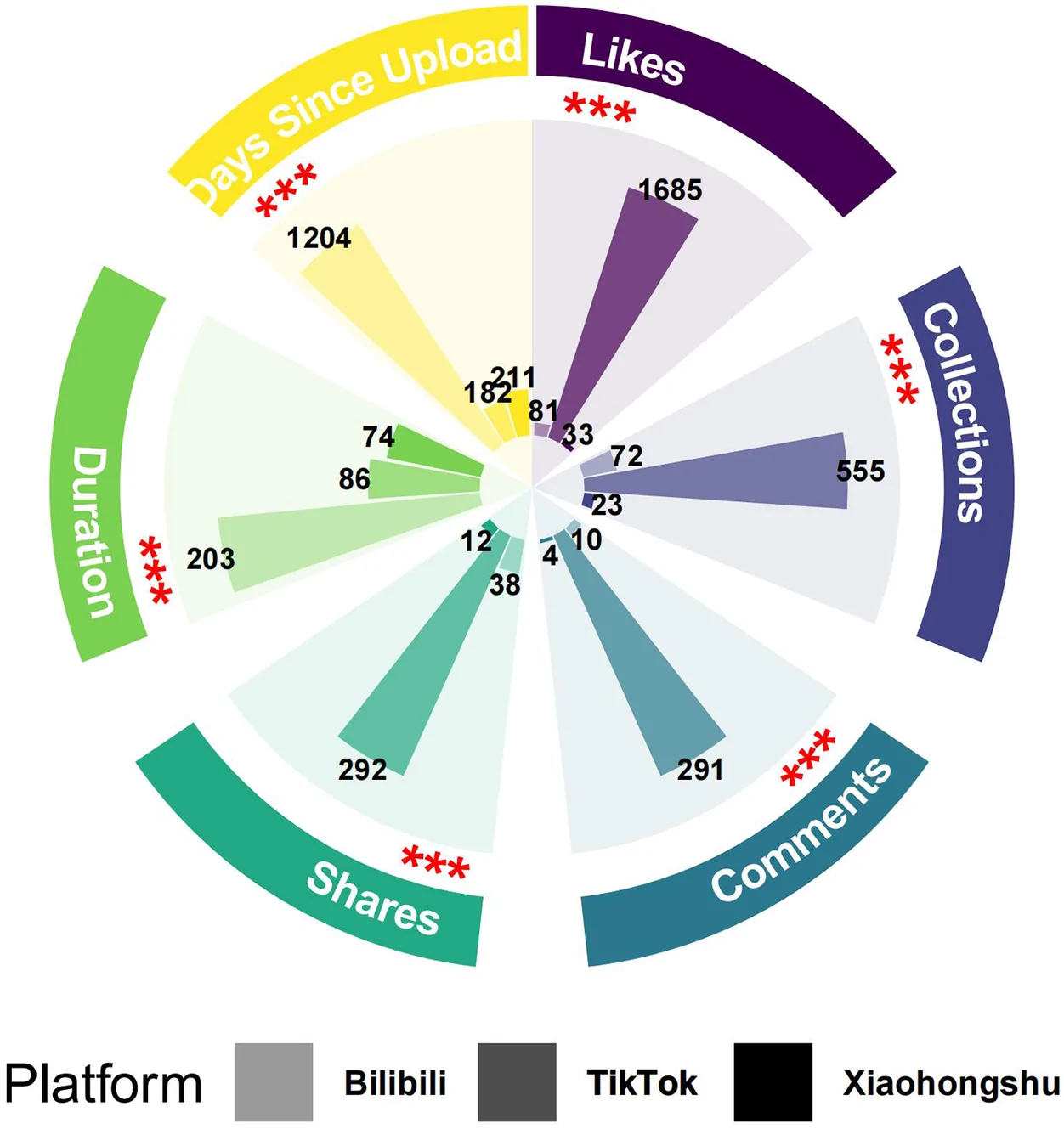
Comparison of engagement metrics and video characteristics across platforms.

Bilibili videos were generally longer and had been available online for a longer period. The median video duration on Bilibili was 203 s, compared with 86 s on TikTok and 74 s on Xiaohongshu. The median days since upload was also highest on Bilibili at 1,204 days, compared with 182 days on TikTok and 211 days on Xiaohongshu. These results indicate clear differences in video duration and time since upload across platforms (Table 1 and Figure 2).

Regarding uploader type, medical personnel were the largest creator group, accounting for 63.67% of all videos, followed by normal users (27.33%), medical institutions (7.00%), and other organizations (2.00%). This suggests that individual medical personnel were the most common source of hepatitis B-related videos in the study sample (Figure 3).

**Figure 3:**
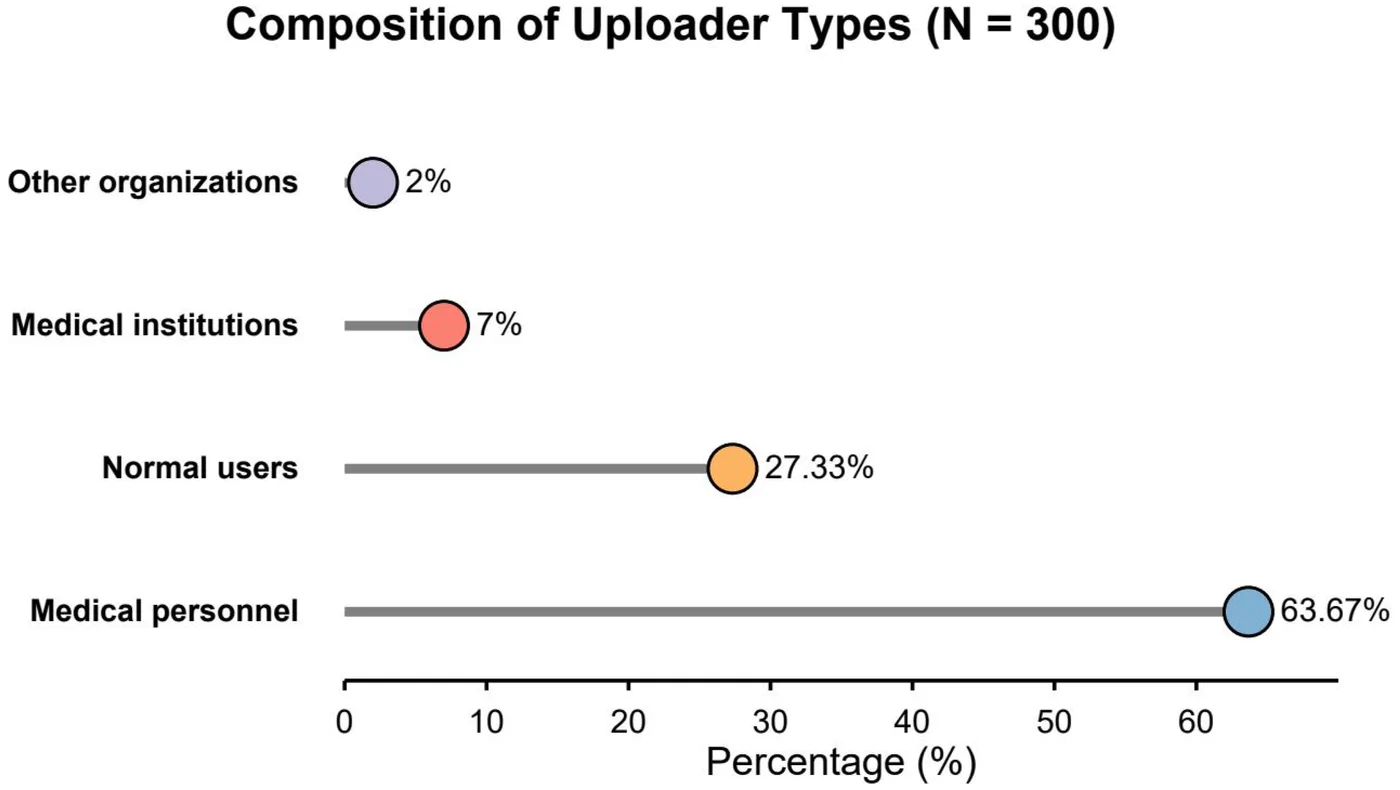
Distribution of uploader types (*N* = 300).

Thematic analysis showed that treatment and prevention were the most common topics, accounting for 64.0% and 56.33% of videos, respectively. The distribution of symptom- and prognosis-related videos did not differ significantly across platforms (*P* > 0.05). However, causes, prevention, and treatment videos showed significant platform differences (*P* < 0.05). For example, Bilibili had a higher proportion of videos addressing HBV causes and transmission. These findings show that hepatitis B-related video content varied across platforms (Table 1 and Figure 4).

**Figure 4:**
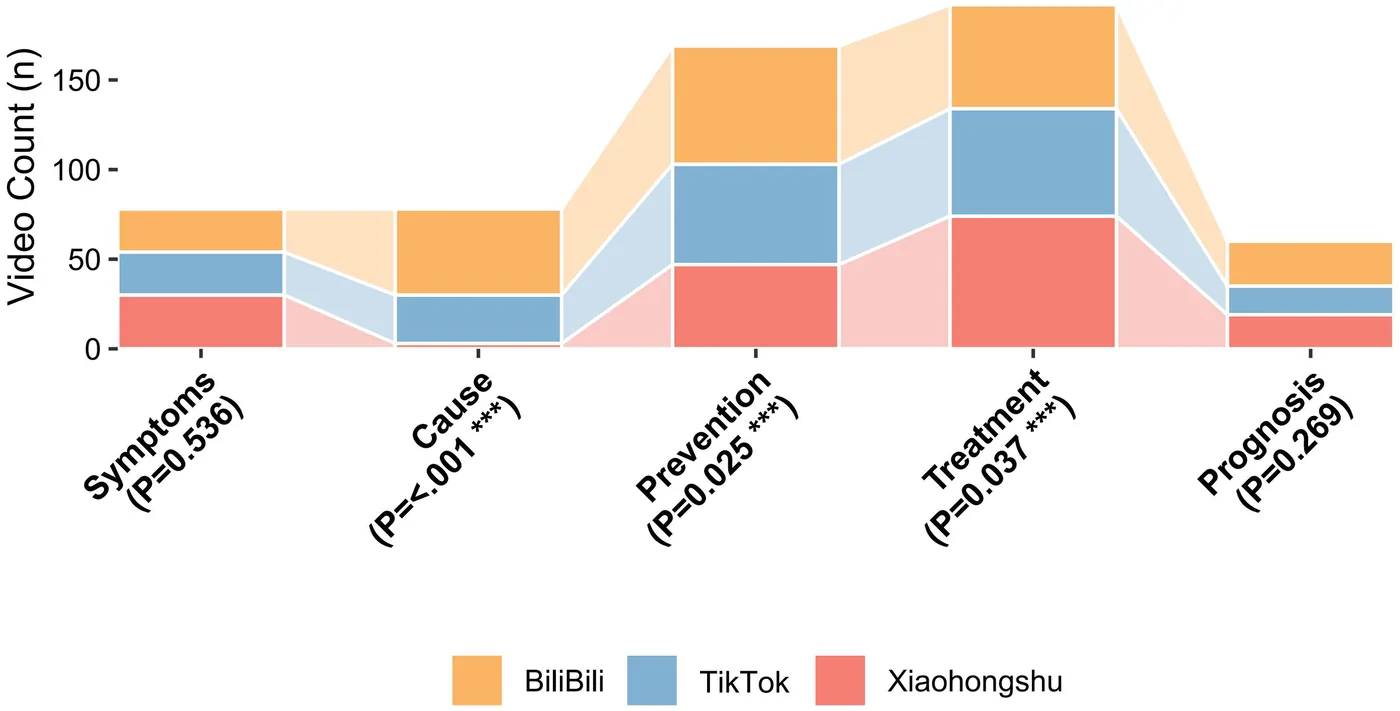
Distribution of video topics across platforms.

### 3.2 Video quality and reliability assessment

Inter-rater agreement varied across the three instruments. Overall weighted agreement was substantial for GQS (*κ* = 0.774, 95% CI 0.705-0.839), almost perfect for mDISCERN (*κ* = 0.928, 95% CI 0.888-0.962), and moderate for JAMA (*κ* = 0.503, 95% CI 0.410-0.590). Platform-specific results are presented in Supplementary Table S2.

GQS, mDISCERN, and JAMA scores differed significantly across uploader types (GQS: H = 11.82, df = 3, *P* = 0.008, *ε*^2^ = 0.030; mDISCERN: H = 9.81, df = 3, *P* = 0.020, *ε*^2^ = 0.023; JAMA: H = 35.69, df = 3, *P* < 0.001, *ε*^2^ = 0.110) (Table 2 and Figure 5). Dunn-Holm comparisons showed that videos uploaded by medical personnel had higher rank distributions than those uploaded by normal users for all three instruments (adjusted *P* = 0.018 for GQS, adjusted *P* = 0.014 for mDISCERN, and adjusted *P* < 0.001 for JAMA). No other uploader-type comparison remained statistically significant after Holm adjustment.

**Table 2:** Comparison of GQS, mDISCERN, and JAMA scores across uploader types.

Variables	Total (*n* = 300)	Medical personnel (*n* = 191)	Normal users (*n* = 82)	Medical institutions (*n* = 21)	Other organizations (*n* = 6)	H	df	*P*	*ε* ^2^
GQS, median (Q1, Q3)	4.00 (4.00, 4.00)	4.00 (4.00, 4.00)	4.00 (3.25, 4.00)	4.00 (3.00, 4.00)	4.00 (3.25, 4.75)	11.82	3	0.008	0.030
mDISCERN, median (Q1, Q3)	4.00 (3.00, 4.00)	4.00 (4.00, 4.00)	4.00 (3.00, 4.00)	4.00 (3.00, 4.00)	3.50 (3.00, 4.75)	9.81	3	0.020	0.023
JAMA, median (Q1, Q3)	3.00 (3.00, 3.00)	3.00 (3.00, 3.00)	3.00 (2.00, 3.00)	3.00 (2.00, 3.00)	3.00 (3.00, 3.00)	35.69	3	<0.001	0.110

Uploader type: medical personnel, normal users, medical institutions, and other organizations. Data are presented as median (Q1, Q3).

**Figure 5:**
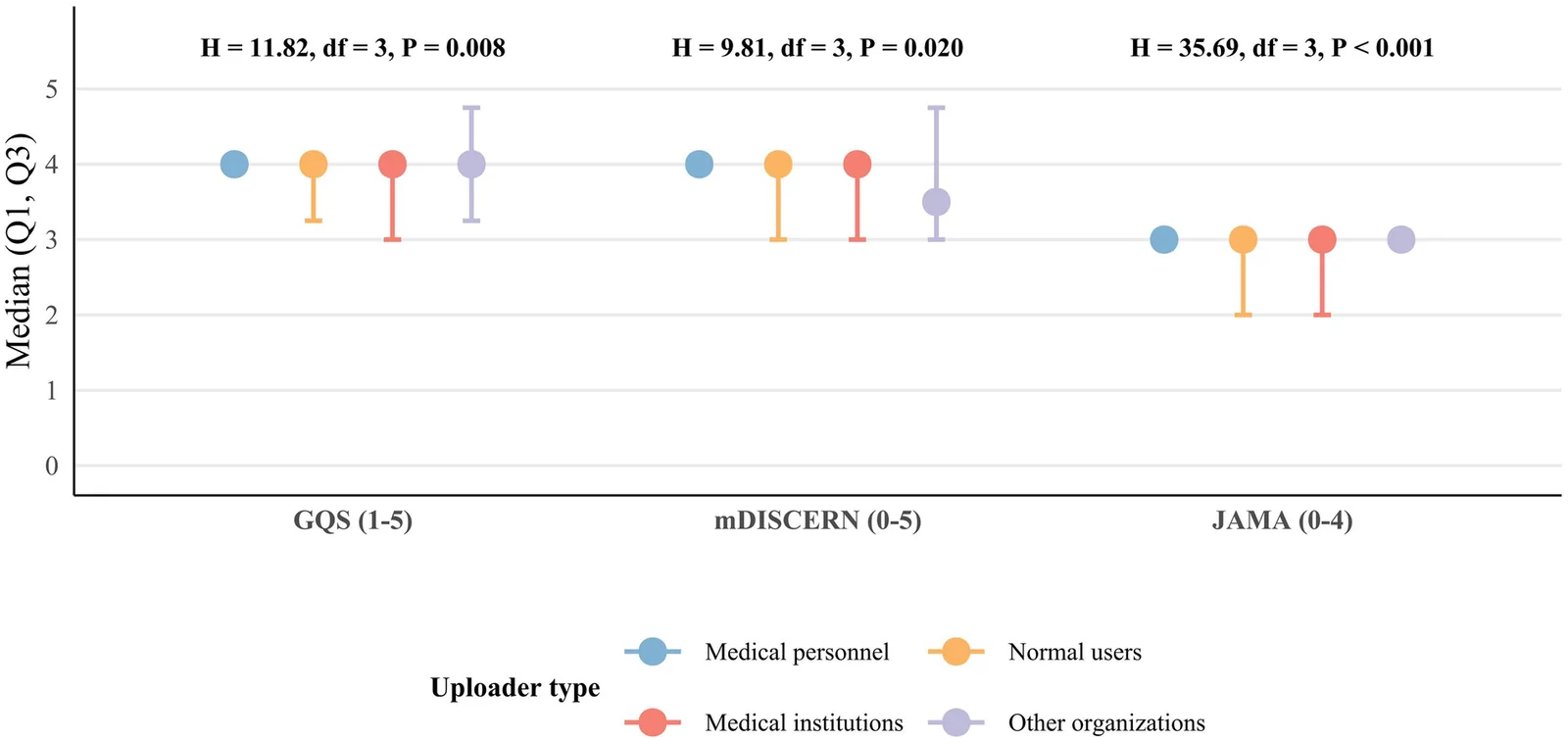
Final consensus quality scores by uploader type. Points represent group medians, and error bars represent interquartile ranges. Uploader-type groups differed substantially in sample size.

Across platforms, GQS scores did not differ significantly (H = 4.61, df = 2, *P* = 0.100, *ε*^2^ = 0.009), whereas mDISCERN (H = 34.60, df = 2, *P* < 0.001, *ε*^2^ = 0.110) and JAMA (H = 24.09, df = 2, *P* < 0.001, *ε*^2^ = 0.074) differed significantly (Table 3 and Figure 6). Dunn-Holm comparisons showed that Xiaohongshu had higher mDISCERN rank distributions than both Bilibili and TikTok. For JAMA, TikTok and Xiaohongshu both had higher rank distributions than Bilibili, whereas TikTok and Xiaohongshu did not differ significantly.

**Table 3:** Platform-based comparison of GQS, mDISCERN, and JAMA scores.

Variables	Total (*n* = 300)	Bilibili (*n* = 100)	TikTok (*n* = 100)	Xiaohongshu (*n* = 100)	H	df	*P*	ε^2^
GQS, median (Q1, Q3)	4.00 (4.00, 4.00)	4.00 (4.00, 4.00)	4.00 (4.00, 4.00)	4.00 (4.00, 4.00)	4.61	2	0.100	0.009
mDISCERN, median (Q1, Q3)	4.00 (3.00, 4.00)	4.00 (3.00, 4.00)	4.00 (3.00, 4.00)	4.00 (4.00, 5.00)	34.60	2	<0.001	0.110
JAMA, median (Q1, Q3)	3.00 (3.00, 3.00)	3.00 (2.00, 3.00)	3.00 (3.00, 3.00)	3.00 (3.00, 3.00)	24.09	2	<0.001	0.074

**Figure 6:**
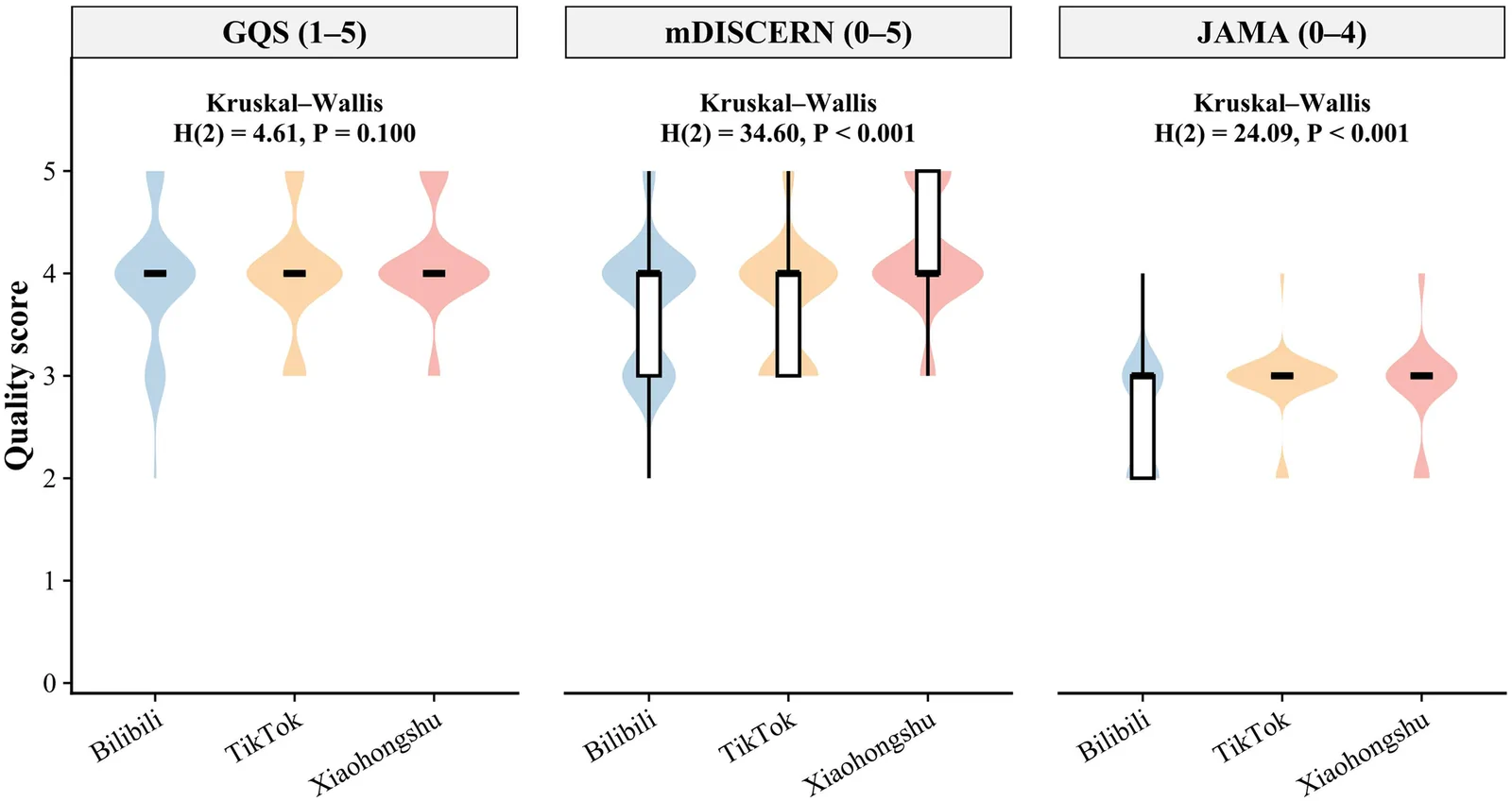
Distribution of final consensus GQS, mDISCERN, and JAMA scores across platforms. Violin plots show the score distributions, and embedded box plots indicate medians and interquartile ranges. Overall differences were assessed using the Kruskal–Wallis H test. Detailed Dunn-Holm pairwise comparisons are reported in Supplementary Table S3.

No statistically significant differences in GQS, mDISCERN, or JAMA scores were observed across presentation formats (GQS: H = 1.89, *P* = 0.389; mDISCERN: H = 0.31, *P* = 0.857; JAMA: H = 5.90, *P* = 0.052) (Table 4). Because the professional-course or lecture group and the personal or shared experience group included only 3 and 4 videos, respectively, these results should be interpreted cautiously.

**Table 4:** Comparison of GQS, mDISCERN, and JAMA scores across presentation formats.

Variables	Total (*n* = 300)	Science popularization/explanatory content (*n* = 293)	Professional courses or lectures (*n* = 3)	Personal or shared experiences (*n* = 4)	H	df	*P*	ε^2^
GQS, median (Q1, Q3)	4.00 (4.00, 4.00)	4.00 (4.00, 4.00)	4.00 (4.00, 4.50)	4.00 (3.75, 4.00)	1.89	2	0.389	0.000
mDISCERN, median (Q1, Q3)	4.00 (3.00, 4.00)	4.00 (3.00, 4.00)	4.00 (4.00, 4.00)	4.00 (3.75, 4.00)	0.31	2	0.857	0.000
JAMA, median (Q1, Q3)	3.00 (3.00, 3.00)	3.00 (3.00, 3.00)	3.00 (2.50, 3.00)	2.00 (2.00, 2.25)	5.90	2	0.052	0.013

Presentation format: science popularization/explanatory content, professional courses or lectures, and personal or shared experiences. Data are presented as median (Q1, Q3). The latter two groups were very small and should be interpreted cautiously.

### 3.3 Spearman correlation analysis

Spearman rank correlation analyses were used to examine unadjusted associations between video characteristics and the final consensus quality scores (Table 5 and Figure 7). These analyses were exploratory and should not be interpreted as independent prediction or causality.

**Table 5:** Spearman correlations between final consensus quality scores and video characteristics.

**Quality score**	**Video characteristic**	***ρ***	**P**
**GQS**	**Likes**	**0**.**166**	**0**.**004**
GQS	Collections	0.145	0.012
GQS	Comments	0.140	0.015
GQS	Shares	0.105	0.070
GQS	Video duration	0.079	0.173
GQS	Days since upload	0.057	0.324
GQS	Followers	0.156	0.007
mDISCERN	Likes	0.023	0.694
mDISCERN	Collections	0.014	0.809
mDISCERN	Comments	0.022	0.706
mDISCERN	Shares	−0.025	0.662
mDISCERN	Video duration	−0.027	0.635
mDISCERN	Days since upload	−0.007	0.899
mDISCERN	Followers	0.034	0.558
JAMA	Likes	0.084	0.146
JAMA	Collections	0.049	0.394
JAMA	Comments	0.071	0.218
JAMA	Shares	0.011	0.854
JAMA	Video duration	−0.178	0.002
JAMA	Days since upload	−0.153	0.008
JAMA	Followers	0.108	0.063

**Figure 7:**
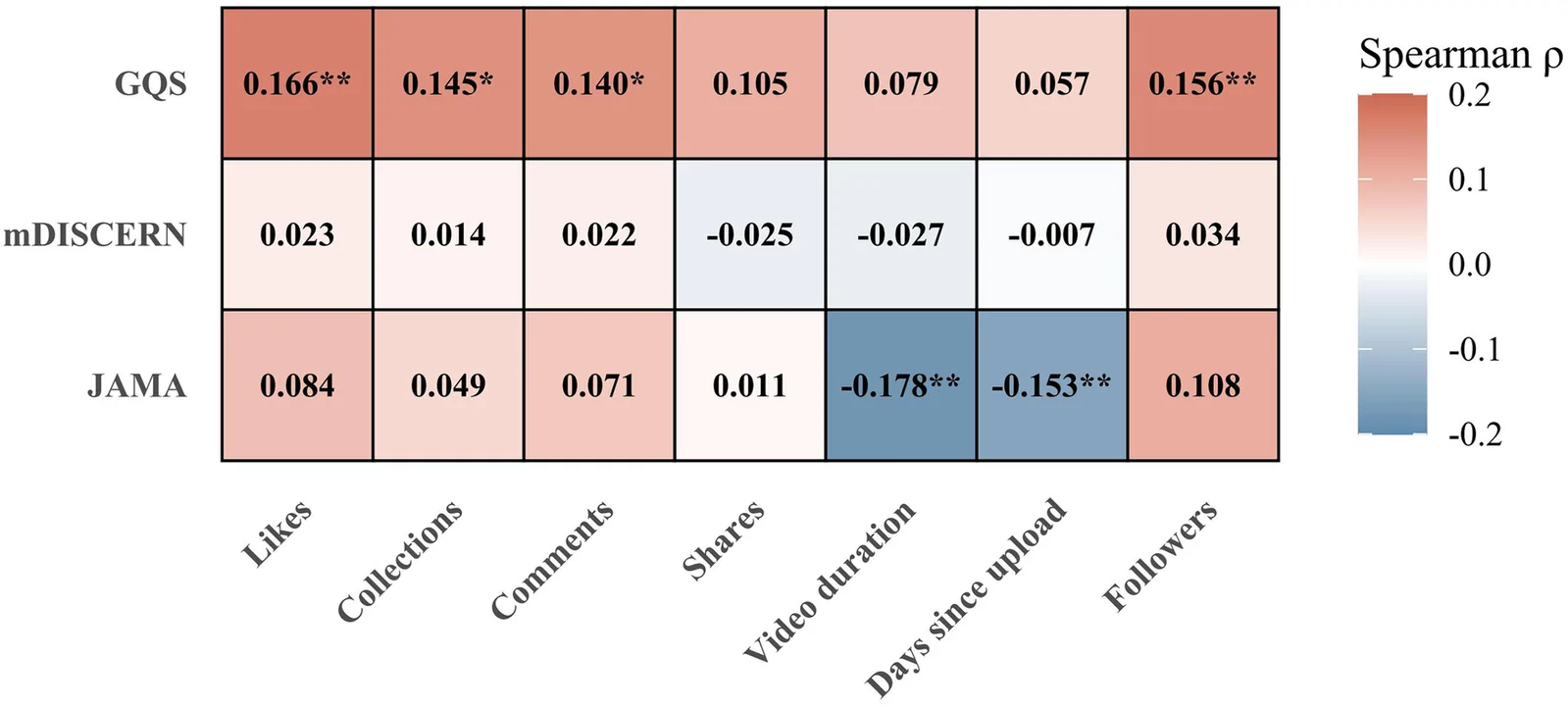
Spearman correlation heatmap of video characteristics and final consensus quality scores. Color intensity represents Spearman *ρ*. Asterisks indicate statistical significance; *P* values were not adjusted for multiple comparisons. The correlations represent exploratory univariable associations.

GQS showed weak positive correlations with likes (*ρ* = 0.166, *P* = 0.004), collections (*ρ* = 0.145, *P* = 0.012), comments (*ρ* = 0.140, *P* = 0.015), and follower count (*ρ* = 0.156, *P* = 0.007). The correlations of GQS with shares, video duration, and days since upload were not statistically significant.

mDISCERN was not significantly correlated with any examined video characteristic. JAMA showed weak negative correlations with video duration (*ρ* = −0.178, *P* = 0.002) and days since upload (*ρ* = −0.153, *P* = 0.008), whereas its correlations with engagement indicators and follower count were not statistically significant.

Overall, engagement indicators showed weak and inconsistent correlations with quality scores. Higher engagement should therefore not be interpreted as evidence of higher reliability or transparency.

### 3.4 Multivariable ordinal regression analysis

In multivariable ordinal regression analyses (Table 6), GQS showed common adjusted associations with platform, uploader type, and video duration. Compared with Bilibili, Xiaohongshu was associated with higher odds of higher GQS scores (aOR = 3.33, 95% CI 1.49-7.45; *P* = 0.003), whereas TikTok was not (aOR = 1.05, 95% CI 0.50-2.21; *P* = 0.897). Compared with medical personnel, normal users were associated with lower odds of higher GQS scores (aOR = 0.37, 95% CI 0.18-0.73; *P* = 0.004), whereas medical institutions and other organizations were not statistically significant. Video duration was positively associated with higher GQS scores (aOR = 1.40 per doubling, 95% CI 1.10-1.79; *P* = 0.007). Follower count showed a threshold-specific association with GQS, reaching statistical significance only for Score >4 (aOR = 1.28, 95% CI 1.14-1.44; *P* < 0.001). Model diagnostics and threshold-specific estimates are shown in Supplementary Table S4 and Supplementary Table S5. Extended topic-adjusted models are summarized in Supplementary Table S6. For mDISCERN, uploader type violated the proportional-odds assumption, and a stable cumulative partial proportional-odds model could not be obtained because of sparse observations in some uploader and score-category combinations, including only one video with an mDISCERN score of 2. Adjusted cumulative associations for mDISCERN were therefore not formally interpreted. A mean bias-reduced adjacent-category ordinal logistic regression was conducted as an exploratory sensitivity analysis, with results presented in Supplementary Table S7. For JAMA, platform, uploader type, and video duration showed threshold-specific associations. TikTok and Xiaohongshu were associated with higher odds of exceeding the Score >2 threshold compared with Bilibili, but these associations were not significant at the Score >3 threshold. Normal users were associated with lower odds of exceeding the Score >2 threshold compared with medical personnel (aOR = 0.22, 95% CI 0.11-0.44; *P* < 0.001), but not at the Score >3 threshold. Video duration was positively associated with the Score >3 threshold (aOR = 1.63, 95% CI 1.03-2.61; *P* = 0.039), but not with Score >2. The extended mDISCERN cumulative model remained unstable and was not formally interpreted.

**Table 6:** Multivariable ordinal regression analyses of factors associated with video quality scores.

Panel A. GQS					
Predictor	Category or unit	Common aOR	95% CI	P	Global P
Platform	Bilibili	Reference			0.001
Platform	TikTok vs. Bilibili	1.05	0.50–2.21	0.897	
Platform	Xiaohongshu vs. Bilibili	3.33	1.49–7.45	0.003	
Uploader type	Medical personnel	Reference			0.019
Uploader type	Normal users vs. Medical personnel	0.37	0.18–0.73	0.004	
Uploader type	Medical institutions vs. Medical personnel	0.41	0.15–1.10	0.077	
Uploader type	Other organizations vs. Medical personnel	0.58	0.09–3.86	0.571	
Continuous characteristics	Follower count, per doubling	Non-proportional effect	See Supplementary Table S5		
Continuous characteristics	Video duration, per doubling	1.40	1.10–1.79	0.007	
Continuous characteristics	Days since upload, per doubling	1.10	0.98–1.23	0.121	

Panel B. mDISCERN					
Predictor	Category or unit	Common aOR	95% CI	P	Global P
mDISCERN cumulative aORs were not formally interpreted because uploader type violated the proportional-odds assumption and sparse uploader-score combinations made the cumulative partial proportional-odds model unstable. Exploratory bias-reduced adjacent-category results are reported in Supplementary Table S7.					

Panel C. JAMA					
Predictor	Category or unit	Common aOR	95% CI	P	Global P
Platform	Bilibili	Reference			0.014
Platform	TikTok vs. Bilibili	Non-proportional effect	See Supplementary Table S5		
Platform	Xiaohongshu vs. Bilibili	Non-proportional effect	See Supplementary Table S5		
Uploader type	Medical personnel	Reference			0.002
Uploader type	Normal users vs. Medical personnel	Non-proportional effect	See Supplementary Table S5		
Uploader type	Medical institutions vs. Medical personnel	Non-proportional effect	See Supplementary Table S5		
Uploader type	Other organizations vs. Medical personnel	Non-proportional effect	See Supplementary Table S5		
Continuous characteristics	Follower count, per doubling	1.01	0.94–1.09	0.788	
Continuous characteristics	Video duration, per doubling	Non-proportional effect	See Supplementary Table S5		
Continuous characteristics	Days since upload, per doubling	1.07	0.95–1.22	0.267	

GQS and JAMA were analyzed using partial proportional-odds cumulative logit models. Common adjusted odds ratios are shown only for predictors satisfying the proportional-odds assumption; threshold-specific estimates for predictors with non-proportional effects are presented in Supplementary Table S5. For mDISCERN, uploader type violated the proportional-odds assumption, and a stable cumulative partial proportional-odds model could not be obtained because of sparse observations in some uploader and score-category combinations. A mean bias-reduced adjacent-category ordinal logistic regression was therefore performed as a sensitivity analysis, with results presented in Supplementary Table S7. Adjusted odds ratios greater than 1 in the cumulative logit models indicate higher odds of being classified into a higher score category. Bilibili and medical personnel served as reference categories.

aOR, adjusted odds ratio; CI, confidence interval; GQS, Global Quality Scale; mDISCERN, modified DISCERN instrument; JAMA, Journal of the American Medical Association benchmark criteria.

## 4 Discussion

### 4.1 Principal findings

This study showed that the apparent quality of hepatitis B-related short videos depended on both the assessment instrument and the source of the content. Platform differences were evident for reliability and transparency, as measured by mDISCERN and JAMA, but not for the unadjusted overall GQS comparison. Differences between medical personnel and normal users were observed across all three instruments in the *post hoc* analyses, whereas the adjusted associations varied by outcome and score threshold. Engagement metrics were only weakly and inconsistently correlated with quality.

The divergence between the instruments is clinically relevant rather than contradictory. GQS mainly reflects overall quality, flow, comprehensiveness, and usefulness; mDISCERN emphasizes clarity of aims, reliability of sources, balance and bias, additional sources, and acknowledgement of uncertainty; and JAMA focuses on authorship, attribution, disclosure, and currency (17–20). A short video may be easy to understand and well organized, thereby receiving a favorable GQS score, while still omitting source attribution, uncertainty, disclosure, or updating information required by mDISCERN and JAMA. This distinction helps explain why platform differences were detected for mDISCERN and JAMA but not for the overall unadjusted GQS comparison. It also indicates that comprehensibility, evidence reliability, and transparency are separate quality dimensions that should not be collapsed into a single judgment.

### 4.2 Platform algorithms, visibility, and instrument-specific platform differences

The use of newly created accounts reduced history-based personalization, but it did not make search retrieval algorithmically neutral. Short-video platforms use proprietary ranking and recommendation systems, and public-health research on TikTok and social media has emphasized the need to examine how platform design and recommendation dynamics shape exposure to health content (21, 22). In this study, the sample should therefore be understood as a snapshot of highly visible videos returned under a specific date, keyword, account-status, and default-ranking condition. Algorithm-related selection could have overrepresented or underrepresented particular uploader types, topics, upload dates, engagement profiles, or quality levels, but the direction and magnitude of this potential bias could not be quantified.

In the present study, mDISCERN and JAMA scores differed across platforms, whereas the overall unadjusted GQS comparison was not significant. Previous studies of short-video health information have reported platform- or source-dependent differences in information quality, but the direction and consistency of these differences vary across diseases, platforms, and assessment instruments (13–15).

The platform-specific findings may partly reflect differences in content ecology, uploader composition, video length, verification practices, and conventions for displaying authorship and source information. These mechanisms were not directly assessed in the present study and remain plausible explanations rather than demonstrated causes. Because JAMA rewards authorship, attribution, disclosure, and currency (20), platform differences in the visibility of such elements could influence transparency scores even when overall educational flow appears similar.

The higher unadjusted mDISCERN rank distributions on Xiaohongshu raise an important question about the relationship between experiential narratives and epistemic authority. Conventional credibility models often privilege formal institutional affiliation and professional credentials (23), but online patient and lifestyle-oriented narratives may contribute situated knowledge by describing how illness, treatment, stigma, uncertainty, and self-management are experienced in everyday life (24–26). Such experiential knowledge may improve relevance and contextual understanding, but it does not replace clinical evidence or establish factual accuracy. The degree of experiential narration was not directly coded in this study, and the adjusted cumulative mDISCERN model was not formally interpreted; therefore, this explanation should be treated as a plausible interpretation rather than a demonstrated mechanism.

Because mDISCERN rewards explicit aims, source cues, balance, additional resources, and acknowledgement of uncertainty, a conversational or experiential format could theoretically perform well when creators clearly delimit personal experience, distinguish it from general medical advice, and provide supporting sources. This possibility was not directly tested in the present study.

The absence of a significant unadjusted platform difference for GQS and the adjusted association between Xiaohongshu and higher GQS scores should be interpreted as answering different questions rather than as conflicting results. The Kruskal–Wallis test compared overall unadjusted rank distributions across platforms, whereas the ordinal regression model adjusted for uploader type, follower count, video duration, and days since upload. One plausible explanation is that additional duration provides more space for structured and comprehensive explanations, although this mechanism was not directly examined in the present study. Longer videos do not necessarily improve authorship disclosure, source attribution, conflict disclosure, or currency, because these transparency elements are separate from narrative length.

### 4.3 Why hepatitis B information is difficult to compress into short videos

Hepatitis B presents a public health communication challenge that is distinct from many acute or symptom-driven conditions. Chronic HBV infection can remain clinically silent for years, and treatment decisions cannot be made on symptoms alone (2, 27–29). Appropriate management requires laboratory-based staging and longitudinal evaluation, including HBsAg testing, HBV DNA assessment, liver function tests, fibrosis assessment, antiviral-treatment eligibility, adherence to long-term therapy, monitoring during treatment, and surveillance for cirrhosis and hepatocellular carcinoma (1, 27–29). These multi-step decisions are difficult to communicate fully in very short videos, especially when content is simplified into binary messages such as positive versus negative, symptomatic versus asymptomatic, or medication versus no medication.

This complexity means that incomplete online explanations may have consequences beyond general health literacy. A video that encourages testing or vaccination may be beneficial, but advice about treatment eligibility, medication discontinuation, pregnancy management, or liver cancer surveillance requires careful qualification (1, 27–29). In a condition characterized by gaps between infection, diagnosis, linkage to care, and treatment, simplified messages could potentially reinforce misunderstandings such as the belief that absence of symptoms means no need for testing, that normal liver function eliminates the need for follow-up, that short-term medication can be stopped independently, or that all HBsAg-positive individuals require immediate treatment. These examples are public health concerns relevant to hepatitis B communication; they were not directly measured as misinformation events in the present sample.

### 4.4 Uploader type, professional expertise, and patient experience

Videos uploaded by medical personnel had higher rank distributions than those uploaded by normal users for all three instruments in the present study. Previous short-video studies have reported source-dependent differences in information quality. Some studies observed higher quality or reliability among professionally produced content, whereas others found no consistent advantage across all instruments or health topics (13–15).

The observed uploader-type pattern is plausible in the context of HBV care. Medical personnel are more likely to be familiar with transmission routes, serological testing, HBV DNA interpretation, antiviral-treatment criteria, long-term monitoring, cirrhosis and liver cancer risk, and pregnancy-related prevention (1, 27–29). Nevertheless, professional identity does not automatically guarantee complete source citation, disclosure, or updating, which may help explain why associations differed across instruments and why JAMA effects were threshold-specific.

The higher quality-rank distributions observed for medical personnel should not be interpreted as evidence that professional identity alone guarantees reliable communication. Medical credentials confer symbolic and epistemic authority, which may increase perceived credibility and persuasiveness, although evidence regarding sharing behavior remains inconsistent (30, 31). This authority can improve public communication when accompanied by evidence-based explanation, but it may also magnify the consequences of incomplete source attribution, insufficient acknowledgement of uncertainty, unbalanced treatment discussion, outdated guidance, or undisclosed commercial interests (32, 33). Professional status should therefore increase accountability rather than exempt content from scrutiny. The relevant policy distinction is not simply between professional and nonprofessional uploaders, but between credential verification and content-level quality assurance.

Normal-user videos may offer lived-experience narratives that differ from professional educational content; however, this potential function was not assessed in the present study (25). Online illness narratives can provide social, emotional, and practical context for patients, but their relevance and applicability may vary across individuals (34, 35). They should therefore be viewed as complementary to, rather than substitutes for, individualized clinical guidance (35). For hepatitis B, a single personal treatment trajectory may not apply to viewers with different viral loads, fibrosis stages, pregnancy status, comorbidities, or treatment histories (1, 27–29). The very small number of videos from other organizations also limits interpretation of that uploader category.

### 4.5 Engagement metrics and information quality

In the present study, engagement metrics were weakly and inconsistently correlated with quality scores. Previous reviews and short-video studies have similarly cautioned that popularity and engagement are unreliable proxies for the accuracy, reliability, or transparency of online health information (7, 10, 11).

Online visibility may be shaped by emotional appeal, controversy, network structure, account popularity, and platform-level distribution mechanisms rather than scientific accuracy alone (10, 11, 36). These mechanisms were not directly measured here, but they offer a plausible explanation for why likes, collections, comments, shares, and follower counts did not consistently track quality. For hepatitis B, where inappropriate interpretation of testing, treatment eligibility, or transmission risk may affect care-seeking and stigma, popularity is an insufficient proxy for information quality.

The mDISCERN modeling results should be interpreted particularly cautiously. The higher unadjusted mDISCERN scores observed on Xiaohongshu remain informative as descriptive platform comparisons, but the adjusted cumulative mDISCERN model was not formally interpreted because uploader type violated the proportional-odds assumption and sparse uploader-score combinations, including only one video with an mDISCERN score of 2, prevented stable estimation. The bias-reduced adjacent-category model in Supplementary Table S7 was therefore exploratory and is not directly comparable with the cumulative odds ratios reported for GQS and JAMA.

### 4.6 Mother-to-child transmission, vaccination, stigma, and transmission misconceptions

Hepatitis B also differs from many other health topics because perinatal and early-childhood infection are central to chronic disease formation. Accurate public communication must distinguish viral transmission from heredity, explain that mother-to-child transmission is preventable, and emphasize antenatal screening, antiviral prophylaxis during pregnancy when indicated, timely birth-dose vaccination, and neonatal immunoprophylaxis (1, 2, 27). Short videos that oversimplify maternal infection as genetic inevitability, or that fail to distinguish infection risk from preventable transmission, could potentially increase fear and stigma rather than promote evidence-based prevention.

Although stigma was not directly assessed in this study, it is an important public health context for interpreting the potential impact of hepatitis B information online. HBV-related stigma in Chinese settings has been linked to misunderstanding of transmission routes, social avoidance, employment and educational discrimination, pressure in intimate relationships, concealment of infection status, and delayed care-seeking (24). Online videos that use fear-based language, blur the distinction between blood/body-fluid transmission and ordinary social contact, or fail to explain vaccination and medical risk stratification may amplify stigma. Conversely, high-quality videos can clarify that hepatitis B is preventable and manageable while also distinguishing genuine transmission risks from casual-contact misconceptions.

### 4.7 Practical implications

Platforms should not rely on professional verification as a proxy for content quality. A two-level governance approach is more appropriate: credential verification should identify who is speaking, whereas content-level standards should assess what is being communicated (37, 38). These standards should apply to all uploaders and should be particularly stringent for high-reach professional accounts because medical authority may amplify both accurate and incomplete messages (32, 37, 38). For hepatitis B videos, content-level standards could include uploader identity and professional qualifications, source or guideline links, posting or updating date, disclosure of commercial interests, intended audience, acknowledgement of uncertainty, and a statement that online content cannot replace individualized clinical consultation.

For high-risk topics such as HBsAg testing, pregnancy management, neonatal vaccination, antiviral treatment, medication discontinuation, and liver cancer surveillance, platforms could provide links to official public health resources or verified medical services (1, 27–29). Health institutions and professional organizations should complement platform moderation through digital-professionalism training, internal review, transparent correction procedures, and guidance on source citation and conflict disclosure. Oversight should be based on content risk and reach rather than on professional identity alone, and professional creators should be encouraged to correct or update incomplete content when guidance changes.

Medical institutions, clinicians, and public health agencies should also adapt evidence-based hepatitis B messages to short-video formats without losing clinical nuance. Effective content should be concise but not misleading, explain key test results and follow-up pathways, correct confusion between heredity and transmission, clarify non-casual transmission routes, reduce stigma, and provide actionable routes to testing and care. Because the study found that engagement metrics are not reliable quality proxies, quality-improvement efforts should not focus only on increasing visibility. Instead, they should aim to improve comprehensibility, reliability, transparency, accountability, and referral value simultaneously.

### 4.8 Limitations and future directions

Several limitations should be noted. First, the cross-sectional design does not support causal inference, and platform mechanisms, algorithmic recommendation processes, viewer knowledge, stigma, care-seeking behavior, and clinical outcomes were not directly measured. Second, videos were sampled using specific search dates and keywords, and results may change as platform algorithms, account status, and health content evolve. Third, engagement indicators may have different meanings across platforms and should not be interpreted as standardized measures of public trust or educational effect. Fourth, some analytic groups were small, including only 6 videos from other organizations and only 3 and 4 videos in two presentation-format categories. Fifth, only one video had an mDISCERN score of 2, and sparse uploader-score combinations prevented stable estimation of a cumulative partial proportional-odds model for mDISCERN; the bias-reduced adjacent-category model was exploratory. Sixth, inter-rater agreement for JAMA was moderate, suggesting that source-transparency judgments may involve greater subjectivity. Finally, unmeasured confounding may remain, and multiple comparisons, threshold-specific models, and exploratory correlations should be interpreted cautiously.

An additional limitation concerns algorithm-related sample selection and limited reproducibility. Each platform applies a proprietary and dynamic ranking system, and relevance could not be defined or harmonized across TikTok, Bilibili, and Xiaohongshu. Although newly created accounts reduced history-based personalization, the retrieved order may still have been influenced by cold-start procedures, geographic or device-level signals, temporal fluctuations, uploader characteristics, engagement patterns, and other undisclosed platform-specific factors. Repeating the same search at another time, location, device, or account could therefore yield a different set or ordering of videos. Such differential ranking could overrepresent or underrepresent particular uploader types, topics, publication dates, or quality levels and may have contributed to the observed cross-platform differences. Because the direction and magnitude of this bias could not be quantified, the findings should be interpreted as describing videos that were highly visible under the specified search conditions rather than all hepatitis B-related content on the platforms.

Future studies should include larger datasets, repeated sampling across multiple dates, cold-start accounts, devices, and geographic contexts, and explicit recording of ranking changes and overlap in retrieved results. Qualitative or mixed-methods research should code narrative style, source citation, balance, uncertainty, factual accuracy, potential harm, and the interaction between professional credentials and lived experience. Additional research should directly assess HBV-specific content accuracy, misinformation themes, stigma-related messaging, viewer comprehension, and the effect of video exposure on testing, vaccination, linkage to care, treatment adherence, and liver cancer surveillance behaviors.

## 5 Conclusion

Among highly visible hepatitis B-related short videos retrieved under the prespecified search conditions, quality varied according to the assessment instrument, platform, and uploader type. Platform differences were observed for mDISCERN and JAMA but not for the overall GQS comparison, while videos uploaded by medical personnel had higher rank distributions across the three instruments than videos uploaded by normal users. Adjusted associations were instrument- and threshold-specific, including patterns involving Xiaohongshu, normal-user uploaders, video duration, and follower count, indicating that no single platform or uploader characteristic was consistently associated with all dimensions of video quality. Engagement metrics were only weakly and inconsistently related to quality and should not be used as proxies for reliability or transparency. These findings support platform-specific quality improvement that emphasizes professional accountability, content-level review, source attribution, information currency, and evidence-based hepatitis B guidance.

## Data Availability

The raw data supporting the conclusions of this article will be made available by the authors, without undue reservation.
