# Supplementary material for: Quality, reliability, and user engagement of hepatitis B–related short videos on TikTok, Bilibili, and Xiaohongshu: a cross-platform cross-sectional study
Source: Front Digit Health. 2026 Oct 9;8:1880030. doi: 10.3389/fdgth.2026.1880030 (PMC13690766; doi:10.3389/fdgth.2026.1880030)
Supplement: Supplementary file 1 [file Table1.docx]

**Supplementary Table S1. GQS, mDISCERN, and JAMA scoring criteria.**

| Instrument | Item or scoring criterion | Score | Interpretation |
| --- | --- | --- | --- |
| GQS | 1. Poor quality and poor flow; most relevant information is missing; not useful for patients. | 1 | Poor quality |
| GQS | 2. Generally poor quality and flow; some information is provided, but several important topics are missing; of very limited usefulness. | 2 | Generally poor quality |
| GQS | 3. Moderate quality; flow is suboptimal; some important information is adequately discussed, but other information is poorly addressed; somewhat useful. | 3 | Moderate quality |
| GQS | 4. Good quality and generally good flow; most relevant information is covered, although some topics are not fully addressed; useful. | 4 | Good quality |
| GQS | 5. Excellent quality and flow; highly comprehensive and useful. | 5 | Excellent quality |
| GQS | Total score range: 1–5. Higher scores indicate better overall quality, flow, and usefulness. |  |  |
| mDISCERN | 1. Are the aims clear and achieved? | 0 or 1 | No = 0; Yes = 1 |
| mDISCERN | 2. Are reliable sources of information used? | 0 or 1 | No = 0; Yes = 1 |
| mDISCERN | 3. Is the information presented in a balanced and unbiased manner? | 0 or 1 | No = 0; Yes = 1 |
| mDISCERN | 4. Are additional sources of information provided? | 0 or 1 | No = 0; Yes = 1 |
| mDISCERN | 5. Are areas of uncertainty acknowledged? | 0 or 1 | No = 0; Yes = 1 |
| mDISCERN | Total score range: 0–5. Higher scores indicate greater reliability and completeness. The five-item mDISCERN instrument used in this study is distinct from the original DISCERN instrument. |  |  |
| JAMA | 1. Authorship: The authors, contributors, affiliations, and relevant credentials are identified. | 0 or 1 | Absent = 0; Present = 1 |
| JAMA | 2. Attribution: References and sources for the information are clearly provided. | 0 or 1 | Absent = 0; Present = 1 |
| JAMA | 3. Disclosure: Ownership, sponsorship, advertising, funding, or potential conflicts of interest are disclosed. | 0 or 1 | Absent = 0; Present = 1 |
| JAMA | 4. Currency: The date of publication or the date of the latest update is provided. | 0 or 1 | Absent = 0; Present = 1 |
| JAMA | Total score range: 0–4. Higher scores indicate greater transparency and reliability of the information source. |  |  |
