# Supplementary material for: Quality, reliability, and user engagement of hepatitis B–related short videos on TikTok, Bilibili, and Xiaohongshu: a cross-platform cross-sectional study
Source: Front Digit Health. 2026 Oct 9;8:1880030. doi: 10.3389/fdgth.2026.1880030 (PMC13690766; doi:10.3389/fdgth.2026.1880030)
Supplement: Supplementary file 2 [file Table2.docx]

**Supplementary Table S2. Inter-rater agreement for GQS, mDISCERN, and JAMA scores by platform**

| **Platform** | **Instrument** | **N** | **Rater 1, median [Q1, Q3]** | **Rater 2, median [Q1, Q3]** | **Exact agreement, n (%)** | **Linearly weighted Cohen’s κ** | **95% CI** | **Interpretation** |
| --- | --- | --- | --- | --- | --- | --- | --- | --- |
| Overall | GQS | 300 | 4 [4, 4] | 4 [4, 4] | 262 (87.3%) | 0.774 | 0.705–0.839 | Substantial |
|  | mDISCERN | 300 | 4 [3, 4] | 4 [3, 4] | 286 (95.3%) | 0.928 | 0.888–0.962 | Almost perfect |
|  | JAMA | 300 | 3 [2, 3] | 3 [3, 3] | 220 (73.3%) | 0.503 | 0.410–0.590 | Moderate |
| Xiaohongshu | GQS | 100 | 4 [4, 4] | 4 [4, 4] | 85 (85.0%) | 0.682 | 0.518–0.819 | Substantial |
|  | mDISCERN | 100 | 4 [4, 5] | 4 [4, 5] | 92 (92.0%) | 0.868 | 0.772–0.948 | Almost perfect |
|  | JAMA | 100 | 3 [2, 3] | 3 [3, 3] | 70 (70.0%) | 0.496 | 0.346–0.636 | Moderate |
| TikTok | GQS | 100 | 4 [3, 4] | 4 [4, 4] | 83 (83.0%) | 0.714 | 0.577–0.829 | Substantial |
|  | mDISCERN | 100 | 4 [3, 4] | 4 [3, 4] | 97 (97.0%) | 0.949 | 0.882–1.000 | Almost perfect |
|  | JAMA | 100 | 3 [3, 3] | 3 [3, 3] | 84 (84.0%) | 0.467 | 0.243–0.668 | Moderate |
| Bilibili | GQS | 100 | 4 [4, 4] | 4 [4, 4] | 94 (94.0%) | 0.899 | 0.814–0.967 | Almost perfect |
|  | mDISCERN | 100 | 4 [3, 4] | 4 [3, 4] | 97 (97.0%) | 0.952 | 0.892–1.000 | Almost perfect |
|  | JAMA | 100 | 2 [2, 3] | 3 [2, 3] | 66 (66.0%) | 0.457 | 0.319–0.593 | Moderate |

Abbreviations: GQS, Global Quality Scale; mDISCERN, modified DISCERN instrument; JAMA, Journal of the American Medical Association benchmark criteria; CI, confidence interval.

Linearly weighted Cohen’s κ was used to assess agreement between the two raters. The 95% confidence intervals were estimated using 5,000 nonparametric bootstrap resamples. For the overall sample, bootstrap resampling was stratified by platform. Exact agreement was defined as the proportion of videos receiving identical total scores from both raters.

Interpretation of κ: <0, poor; 0.00–0.20, slight; 0.21–0.40, fair; 0.41–0.60, moderate; 0.61–0.80, substantial; and 0.81–1.00, almost perfect.
