# Supplementary material for: Quality, reliability, and user engagement of hepatitis B–related short videos on TikTok, Bilibili, and Xiaohongshu: a cross-platform cross-sectional study
Source: Front Digit Health. 2026 Oct 9;8:1880030. doi: 10.3389/fdgth.2026.1880030 (PMC13690766; doi:10.3389/fdgth.2026.1880030)
Supplement: Supplementary file 3 [file Table3.docx]

**Supplementary Table S3. Dunn-Holm post hoc pairwise comparisons.**

| Grouping variable | Instrument | Group 1 | Group 2 | Group 1, n | Group 2, n | Group 1, median (Q1, Q3) | Group 2, median (Q1, Q3) | Mean rank difference | Dunn Z | Raw P | Holm-adjusted P | Significant after Holm adjustment |
| --- | --- | --- | --- | --- | --- | --- | --- | --- | --- | --- | --- | --- |
| Uploader type | GQS | Medical personnel | Normal users | 191 | 82 | 4.00 (4.00, 4.00) | 4.00 (3.25, 4.00) | 27.676 | 2.975 | 0.003 | 0.018 | Yes |
| Uploader type | GQS | Medical personnel | Medical institutions | 191 | 21 | 4.00 (4.00, 4.00) | 4.00 (3.00, 4.00) | 35.822 | 2.211 | 0.027 | 0.135 | No |
| Uploader type | GQS | Medical personnel | Other organizations | 191 | 6 | 4.00 (4.00, 4.00) | 4.00 (3.25, 4.75) | 11.298 | 0.387 | 0.699 | 1.000 | No |
| Uploader type | GQS | Normal users | Medical institutions | 82 | 21 | 4.00 (3.25, 4.00) | 4.00 (3.00, 4.00) | 8.146 | 0.473 | 0.636 | 1.000 | No |
| Uploader type | GQS | Normal users | Other organizations | 82 | 6 | 4.00 (3.25, 4.00) | 4.00 (3.25, 4.75) | -16.378 | -0.55 | 0.583 | 1.000 | No |
| Uploader type | GQS | Medical institutions | Other organizations | 21 | 6 | 4.00 (3.00, 4.00) | 4.00 (3.25, 4.75) | -24.524 | -0.752 | 0.452 | 1.000 | No |
| Uploader type | mDISCERN | Medical personnel | Normal users | 191 | 82 | 4.00 (4.00, 4.00) | 4.00 (3.00, 4.00) | 30.583 | 3.054 | 0.002 | 0.014 | Yes |
| Uploader type | mDISCERN | Medical personnel | Medical institutions | 191 | 21 | 4.00 (4.00, 4.00) | 4.00 (3.00, 4.00) | 19.954 | 1.144 | 0.252 | 1.000 | No |
| Uploader type | mDISCERN | Medical personnel | Other organizations | 191 | 6 | 4.00 (4.00, 4.00) | 3.50 (3.00, 4.75) | 19.394 | 0.617 | 0.537 | 1.000 | No |
| Uploader type | mDISCERN | Normal users | Medical institutions | 82 | 21 | 4.00 (3.00, 4.00) | 4.00 (3.00, 4.00) | -10.63 | -0.573 | 0.567 | 1.000 | No |
| Uploader type | mDISCERN | Normal users | Other organizations | 82 | 6 | 4.00 (3.00, 4.00) | 3.50 (3.00, 4.75) | -11.189 | -0.349 | 0.727 | 1.000 | No |
| Uploader type | mDISCERN | Medical institutions | Other organizations | 21 | 6 | 4.00 (3.00, 4.00) | 3.50 (3.00, 4.75) | -0.56 | -0.016 | 0.987 | 1.000 | No |
| Uploader type | JAMA | Medical personnel | Normal users | 191 | 82 | 3.00 (3.00, 3.00) | 3.00 (2.00, 3.00) | 52.907 | 5.893 | <0.001 | <0.001 | Yes |
| Uploader type | JAMA | Medical personnel | Medical institutions | 191 | 21 | 3.00 (3.00, 3.00) | 3.00 (2.00, 3.00) | 24.243 | 1.551 | 0.121 | 0.363 | No |
| Uploader type | JAMA | Medical personnel | Other organizations | 191 | 6 | 3.00 (3.00, 3.00) | 3.00 (3.00, 3.00) | -6.471 | -0.23 | 0.818 | 0.818 | No |
| Uploader type | JAMA | Normal users | Medical institutions | 82 | 21 | 3.00 (2.00, 3.00) | 3.00 (2.00, 3.00) | -28.664 | -1.724 | 0.085 | 0.339 | No |
| Uploader type | JAMA | Normal users | Other organizations | 82 | 6 | 3.00 (2.00, 3.00) | 3.00 (3.00, 3.00) | -59.378 | -2.065 | 0.039 | 0.195 | No |
| Uploader type | JAMA | Medical institutions | Other organizations | 21 | 6 | 3.00 (2.00, 3.00) | 3.00 (3.00, 3.00) | -30.714 | -0.976 | 0.329 | 0.658 | No |
| Platform | mDISCERN | Bilibili | TikTok | 100 | 100 | 4.00 (3.00, 4.00) | 4.00 (3.00, 4.00) | 2.005 | 0.187 | 0.852 | 0.852 | No |
| Platform | mDISCERN | Bilibili | Xiaohongshu | 100 | 100 | 4.00 (3.00, 4.00) | 4.00 (4.00, 5.00) | -53.605 | -4.998 | <0.001 | <0.001 | Yes |
| Platform | mDISCERN | TikTok | Xiaohongshu | 100 | 100 | 4.00 (3.00, 4.00) | 4.00 (4.00, 5.00) | -55.61 | -5.185 | <0.001 | <0.001 | Yes |
| Platform | JAMA | Bilibili | TikTok | 100 | 100 | 3.00 (2.00, 3.00) | 3.00 (3.00, 3.00) | -43.29 | -4.502 | <0.001 | <0.001 | Yes |
| Platform | JAMA | Bilibili | Xiaohongshu | 100 | 100 | 3.00 (2.00, 3.00) | 3.00 (3.00, 3.00) | -37.935 | -3.945 | <0.001 | <0.001 | Yes |
| Platform | JAMA | TikTok | Xiaohongshu | 100 | 100 | 3.00 (3.00, 3.00) | 3.00 (3.00, 3.00) | 5.355 | 0.557 | 0.578 | 0.578 | No |
