# Supplementary material for: Quality, reliability, and user engagement of hepatitis B–related short videos on TikTok, Bilibili, and Xiaohongshu: a cross-platform cross-sectional study
Source: Front Digit Health. 2026 Oct 9;8:1880030. doi: 10.3389/fdgth.2026.1880030 (PMC13690766; doi:10.3389/fdgth.2026.1880030)
Supplement: Supplementary file 4 [file Table4.docx]

**Supplementary Table S4. Ordinal regression model diagnostics.**

| Outcome | N | Core_model_formula | Final_model_type | Variables_with_non_proportional_effects | Log_likelihood | AIC | BIC | LR_test_comparing_proportional_and_partial_models | Maximum_VIF | Convergence | Hessian_status | Maximum_gradient | Separation_warning | Final_interpretation_status |
| --- | --- | --- | --- | --- | --- | --- | --- | --- | --- | --- | --- | --- | --- | --- |
| GQS | 300 | GQS_final ~ Platform_Display + Uploader_Type + log2_followers + log2_duration + log2_days_since_upload | Partial proportional-odds cumulative logit | log2_followers | -232.16 | 490.32 | 538.47 | LR = 10.95, df = 2, P = 0.004 | 2.12 | Converged | Positive definite | 1.36e-02 | None | Reportable |
| mDISCERN | 300 | mDISCERN_final ~ Platform_Display + Uploader_Type + log2_followers + log2_duration + log2_days_since_upload | Cumulative partial proportional-odds model not formally interpretable; bias-reduced adjacent-category model performed as a sensitivity analysis | Uploader_Type | -248.25 | 530.49 | 593.46 | LR = 13.87, df = 6, P = 0.031 | 2.12 | Converged | Covariance matrix available; cumulative threshold-specific CI unstable at sparse Score > 2 threshold | Not available from VGAM vglm; coefficient finiteness checked | Sparse mDISCERN score category: score 2, n = 1 | Not formally interpretable |
| JAMA | 300 | JAMA_final ~ Platform_Display + Uploader_Type + log2_followers + log2_duration + log2_days_since_upload | Partial proportional-odds cumulative logit | Platform_Display; Uploader_Type; log2_duration | -184.70 | 401.39 | 460.65 | LR = 18.65, df = 6, P = 0.005 | 2.12 | Converged | Positive definite | 8.39e-04 | None | Reportable |
