# Supplementary material for: Quality, reliability, and user engagement of hepatitis B–related short videos on TikTok, Bilibili, and Xiaohongshu: a cross-platform cross-sectional study
Source: Front Digit Health. 2026 Oct 9;8:1880030. doi: 10.3389/fdgth.2026.1880030 (PMC13690766; doi:10.3389/fdgth.2026.1880030)
Supplement: Supplementary file 5 [file Table5.docx]

**Supplementary Table S5. Threshold-specific estimates for non-proportional effects.**

| Outcome | Predictor | Comparison | Cumulative_threshold | aOR | CI_95 | P | Model_convergence | Estimate_stability |
| --- | --- | --- | --- | --- | --- | --- | --- | --- |
| GQS | Continuous characteristics | Follower count, per doubling | Score > 2 | 0.98 | 0.73-1.32 | 0.906 | Converged | Stable finite estimate |
| GQS | Continuous characteristics | Follower count, per doubling | Score > 3 | 1.03 | 0.94-1.12 | 0.549 | Converged | Stable finite estimate |
| GQS | Continuous characteristics | Follower count, per doubling | Score > 4 | 1.28 | 1.14-1.44 | <0.001 | Converged | Stable finite estimate |
| JAMA | Platform | TikTok vs Bilibili | Score > 2 | 3.97 | 1.58-9.98 | 0.003 | Converged | Stable finite estimate |
| JAMA | Platform | TikTok vs Bilibili | Score > 3 | 1.13 | 0.19-6.76 | 0.891 | Converged | Stable finite estimate |
| JAMA | Platform | Xiaohongshu vs Bilibili | Score > 2 | 2.41 | 1.02-5.69 | 0.045 | Converged | Stable finite estimate |
| JAMA | Platform | Xiaohongshu vs Bilibili | Score > 3 | 3.16 | 0.71-14.10 | 0.132 | Converged | Stable finite estimate |
| JAMA | Uploader type | Normal users vs Medical personnel | Score > 3 | 0.67 | 0.15-2.98 | 0.600 | Converged | Stable finite estimate |
| JAMA | Uploader type | Normal users vs Medical personnel | Score > 2 | 0.22 | 0.11-0.44 | <0.001 | Converged | Stable finite estimate |
| JAMA | Uploader type | Medical institutions vs Medical personnel | Score > 3 | 1.05 | 0.12-9.40 | 0.969 | Converged | Stable finite estimate |
| JAMA | Uploader type | Medical institutions vs Medical personnel | Score > 2 | 0.33 | 0.11-1.00 | 0.050 | Converged | Stable finite estimate |
| JAMA | Uploader type | Other organizations vs Medical personnel | Score > 2 | 1.08 | 0.11-10.23 | 0.944 | Converged | Stable finite estimate |
| JAMA | Uploader type | Other organizations vs Medical personnel | Score > 3 | 5.30 | 0.43-64.79 | 0.191 | Converged | Stable finite estimate |
| JAMA | Continuous characteristics | Video duration, per doubling | Score > 2 | 0.91 | 0.68-1.22 | 0.533 | Converged | Stable finite estimate |
| JAMA | Continuous characteristics | Video duration, per doubling | Score > 3 | 1.63 | 1.03-2.61 | 0.039 | Converged | Stable finite estimate |
