# Supplementary material for: Quality, reliability, and user engagement of hepatitis B–related short videos on TikTok, Bilibili, and Xiaohongshu: a cross-platform cross-sectional study
Source: Front Digit Health. 2026 Oct 9;8:1880030. doi: 10.3389/fdgth.2026.1880030 (PMC13690766; doi:10.3389/fdgth.2026.1880030)
Supplement: Supplementary file 6 [file Table6.docx]

**Supplementary Table S6. Extended topic-adjusted models.**

| Outcome | Predictor | Category_or_unit | Common_aOR_95_CI | P | Non_proportional_terms_in_extended_model |
| --- | --- | --- | --- | --- | --- |
| GQS | Platform | TikTok vs Bilibili | 1.28 (0.59-2.77) | 0.530 | log2_followers |
| mDISCERN | Extended topic-adjusted model | Not formally interpreted | The extended topic-adjusted mDISCERN cumulative partial proportional-odds model was not formally interpreted because stable finite estimates could not be obtained. |  | Uploader_Type |
| GQS | Platform | Xiaohongshu vs Bilibili | 4.35 (1.82-10.38) | <0.001 | log2_followers |
| GQS | Uploader type | Normal users vs Medical personnel | 0.39 (0.19-0.77) | 0.007 | log2_followers |
| GQS | Uploader type | Medical institutions vs Medical personnel | 0.38 (0.14-1.04) | 0.059 | log2_followers |
| GQS | Uploader type | Other organizations vs Medical personnel | 0.56 (0.08-3.77) | 0.551 | log2_followers |
| GQS | Continuous characteristics | Video duration, per doubling | 1.37 (1.07-1.75) | 0.012 | log2_followers |
| GQS | Continuous characteristics | Days since upload, per doubling | 1.10 (0.97-1.24) | 0.125 | log2_followers |
| JAMA | Continuous characteristics | Days since upload, per doubling | 1.08 (0.94-1.23) | 0.273 | Platform_Display; Uploader_Type; log2_duration |
| GQS | Video topics | Symptoms: Yes vs No | 1.11 (0.59-2.09) | 0.741 | log2_followers |
| JAMA | Video topics | Symptoms: Yes vs No | 0.83 (0.40-1.71) | 0.608 | Platform_Display; Uploader_Type; log2_duration |
| GQS | Video topics | Cause: Yes vs No | 1.64 (0.80-3.33) | 0.175 | log2_followers |
| JAMA | Video topics | Cause: Yes vs No | 1.72 (0.75-3.95) | 0.199 | Platform_Display; Uploader_Type; log2_duration |
| GQS | Video topics | Prevention: Yes vs No | 1.17 (0.66-2.08) | 0.594 | log2_followers |
| JAMA | Video topics | Prevention: Yes vs No | 1.25 (0.67-2.34) | 0.490 | Platform_Display; Uploader_Type; log2_duration |
| GQS | Video topics | Treatment: Yes vs No | 1.21 (0.65-2.25) | 0.554 | log2_followers |
| JAMA | Video topics | Treatment: Yes vs No | 0.91 (0.45-1.85) | 0.797 | Platform_Display; Uploader_Type; log2_duration |
| GQS | Video topics | Prognosis: Yes vs No | 2.08 (1.02-4.28) | 0.045 | log2_followers |
| JAMA | Continuous characteristics | Follower count, per doubling | 1.01 (0.94-1.09) | 0.797 | Platform_Display; Uploader_Type; log2_duration |
