# Supplementary material for: Quality, reliability, and user engagement of hepatitis B–related short videos on TikTok, Bilibili, and Xiaohongshu: a cross-platform cross-sectional study
Source: Front Digit Health. 2026 Oct 9;8:1880030. doi: 10.3389/fdgth.2026.1880030 (PMC13690766; doi:10.3389/fdgth.2026.1880030)
Supplement: Supplementary file 7 [file Table7.docx]

**Supplementary Table S7. mDISCERN bias-reduced adjacent-category sensitivity analysis.**

| Outcome | Model | Adjacent-category contrast | Predictor | Comparison or unit | aOR | 95% CI | P | Estimate stability |
| --- | --- | --- | --- | --- | --- | --- | --- | --- |
| mDISCERN | Bias-reduced adjacent-category ordinal logistic model | Score 2 vs Score 3 | Platform | TikTok vs Bilibili | 0.56 | 0.03-12.14 | 0.712 | Finite bias-reduced estimate |
| mDISCERN | Bias-reduced adjacent-category ordinal logistic model | Score 3 vs Score 4 | Platform | TikTok vs Bilibili | 1.53 | 0.66-3.52 | 0.322 | Finite bias-reduced estimate |
| mDISCERN | Bias-reduced adjacent-category ordinal logistic model | Score 4 vs Score 5 | Platform | TikTok vs Bilibili | 0.76 | 0.21-2.65 | 0.662 | Finite bias-reduced estimate |
| mDISCERN | Bias-reduced adjacent-category ordinal logistic model | Score 2 vs Score 3 | Platform | Xiaohongshu vs Bilibili | 2.70 | 0.14-51.68 | 0.510 | Finite bias-reduced estimate |
| mDISCERN | Bias-reduced adjacent-category ordinal logistic model | Score 3 vs Score 4 | Platform | Xiaohongshu vs Bilibili | 0.22 | 0.07-0.65 | 0.006 | Finite bias-reduced estimate |
| mDISCERN | Bias-reduced adjacent-category ordinal logistic model | Score 4 vs Score 5 | Platform | Xiaohongshu vs Bilibili | 0.12 | 0.04-0.38 | <0.001 | Finite bias-reduced estimate |
| mDISCERN | Bias-reduced adjacent-category ordinal logistic model | Score 2 vs Score 3 | Uploader type | Normal users vs Medical personnel | 1.24 | 0.09-16.30 | 0.871 | Finite bias-reduced estimate |
| mDISCERN | Bias-reduced adjacent-category ordinal logistic model | Score 3 vs Score 4 | Uploader type | Normal users vs Medical personnel | 3.60 | 1.69-7.66 | <0.001 | Finite bias-reduced estimate |
| mDISCERN | Bias-reduced adjacent-category ordinal logistic model | Score 4 vs Score 5 | Uploader type | Normal users vs Medical personnel | 0.62 | 0.23-1.66 | 0.337 | Finite bias-reduced estimate |
| mDISCERN | Bias-reduced adjacent-category ordinal logistic model | Score 2 vs Score 3 | Uploader type | Medical institutions vs Medical personnel | 2.67 | 0.12-58.74 | 0.533 | Finite bias-reduced estimate |
| mDISCERN | Bias-reduced adjacent-category ordinal logistic model | Score 3 vs Score 4 | Uploader type | Medical institutions vs Medical personnel | 2.54 | 0.86-7.49 | 0.092 | Finite bias-reduced estimate |
| mDISCERN | Bias-reduced adjacent-category ordinal logistic model | Score 4 vs Score 5 | Uploader type | Medical institutions vs Medical personnel | 0.32 | 0.08-1.26 | 0.102 | Finite bias-reduced estimate |
| mDISCERN | Bias-reduced adjacent-category ordinal logistic model | Score 2 vs Score 3 | Uploader type | Other organizations vs Medical personnel | 3.33 | 0.06-192.98 | 0.562 | Finite bias-reduced estimate |
| mDISCERN | Bias-reduced adjacent-category ordinal logistic model | Score 3 vs Score 4 | Uploader type | Other organizations vs Medical personnel | 10.87 | 1.06-111.23 | 0.044 | Finite bias-reduced estimate |
| mDISCERN | Bias-reduced adjacent-category ordinal logistic model | Score 4 vs Score 5 | Uploader type | Other organizations vs Medical personnel | 0.07 | 0.01-0.94 | 0.045 | Finite bias-reduced estimate |
| mDISCERN | Bias-reduced adjacent-category ordinal logistic model | Score 2 vs Score 3 | Continuous characteristics | Follower count, per doubling | 0.96 | 0.76-1.21 | 0.745 | Finite bias-reduced estimate |
| mDISCERN | Bias-reduced adjacent-category ordinal logistic model | Score 3 vs Score 4 | Continuous characteristics | Follower count, per doubling | 0.96 | 0.89-1.04 | 0.349 | Finite bias-reduced estimate |
| mDISCERN | Bias-reduced adjacent-category ordinal logistic model | Score 4 vs Score 5 | Continuous characteristics | Follower count, per doubling | 0.87 | 0.78-0.98 | 0.019 | Finite bias-reduced estimate |
| mDISCERN | Bias-reduced adjacent-category ordinal logistic model | Score 2 vs Score 3 | Continuous characteristics | Video duration, per doubling | 1.42 | 0.57-3.49 | 0.449 | Finite bias-reduced estimate |
| mDISCERN | Bias-reduced adjacent-category ordinal logistic model | Score 3 vs Score 4 | Continuous characteristics | Video duration, per doubling | 0.89 | 0.66-1.20 | 0.447 | Finite bias-reduced estimate |
| mDISCERN | Bias-reduced adjacent-category ordinal logistic model | Score 4 vs Score 5 | Continuous characteristics | Video duration, per doubling | 0.69 | 0.50-0.96 | 0.027 | Finite bias-reduced estimate |
| mDISCERN | Bias-reduced adjacent-category ordinal logistic model | Score 2 vs Score 3 | Continuous characteristics | Days since upload, per doubling | 1.03 | 0.67-1.58 | 0.897 | Finite bias-reduced estimate |
| mDISCERN | Bias-reduced adjacent-category ordinal logistic model | Score 3 vs Score 4 | Continuous characteristics | Days since upload, per doubling | 0.86 | 0.75-0.99 | 0.039 | Finite bias-reduced estimate |
| mDISCERN | Bias-reduced adjacent-category ordinal logistic model | Score 4 vs Score 5 | Continuous characteristics | Days since upload, per doubling | 1.00 | 0.86-1.15 | 0.953 | Finite bias-reduced estimate |
